# Sodium chloride stress inhibits *Saccharomyces paradoxus* meiosis

**DOI:** 10.17912/micropub.biology.002115

**Published:** 2026-05-05

**Authors:** Gemima Germain, Sylvia Lamphere, Ella Molinari, Primrose Boynton

**Affiliations:** 1 Department of Biological, Chemical, and Environmental Sciences, Wheaton College - Massachusetts, Norton, MA, US

## Abstract

Meiosis is a common response to nutrient deprivation in yeasts. Our goal was to determine whether the yeast *Saccharomyces paradoxus* performed meiosis under other abiotic stress factors, specifically salt stress. We predicted that *S. paradoxus* meiosis would increase in the presence of salt because osmotic stress activates the IME1 transcription factor in its model yeast relative *S. cerevisiae* . Contrary to our prediction, the sporulation rate of *S. paradoxus* decreased as salinity increased. We hypothesize that this is due to salt inhibiting mitochondrial function, but more studies are needed to determine the cause.

**Figure 1. S. paradoxus sporulation in different concentrations of salt:** Sporulation efficiency decreases as the amount of salt in the environment increases. A) Photograph of vegetative cells (1) and sporulated tetrads (2). B) Relationship between percent salt and proportion of cells sporulating. Small black points are individual sporulation observations; black lines connect values for the same *S. paradoxus* isolate. Large red points are averages for each category of salt percentage (0, 3%, or 6% sodium chloride).

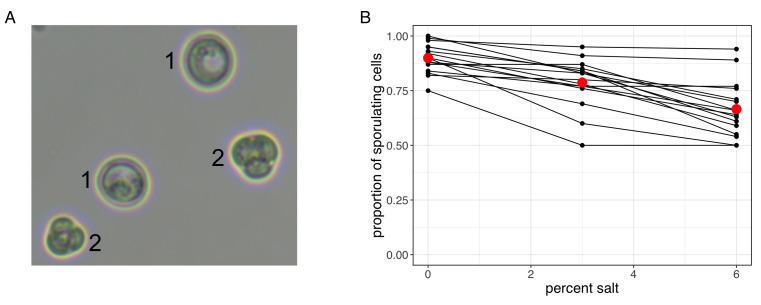



## Description

Sex, the life cycle characterized by meiosis and mating, is facultative for most eukaryotes (Nieuwenhuis and James 2016, Speijer et al. 2015). Sexual life cycles have many costs compared to asexual reproduction, including reduced growth rates, metabolic investments in meiosis and mating, and costs of finding mates, but the machinery for sex is conserved over the eukaryotic tree of life because it is the primary mechanism for eukaryotic genetic recombination (Lehtonen et al. 2012, Speijer et al. 2015). The advantages of sexual recombination, especially in the face of changing environments, can explain its ubiquity (Becks and Agrawal 2012, Lively and Morran, 2014). Sex is favored in changing environments in experimental studies (Morran et al. 2011), and stressful environments often trigger meiosis in facultatively sexual eukaryotes (Ram and Hadany 2016, Schoustra et al. 2010). For example, the facultatively sexual model yeast *Saccharomyces cerevisiae* undergoes meiosis and produces haploid spores (sporulation) in laboratory environments when it is nutrient-starved (Mitchell 1994) ( Figure 1A ), but its wild sister species *S. paradoxus* , which has similar physiology, rarely undergoes meiosis in non-domesticated environments (Tsai et al. 2008, Vaughan-Martini and Martini 2011). We would like to understand if meiosis as a response to stress is generalizable to a non-nutrient-related stressor in *S. paradoxus* .

Similarly to nutrient stress, we hypothesized that salt (sodium chloride) stress might increase *S. paradoxus* meiosis frequency. In *Saccharomyces* , meiosis is induced by the transcription factor IME1 (Vershon and Pierce 2000). In *S. cerevisiae,* signaling proteins in the Hog1 osmotic stress response pathway, triggered by high environmental sodium chloride, bind to regulatory DNA associated with IME1, inducing transcription (Kahana-Edwin et al. 2013). Additionally, salt increases crossovers during meiosis in another model eukaryote, *Arabidopsis thaliana* (van Tol et al. 2018). We hypothesized that these molecular mechanisms are likely to translate to increased *S. paradoxus* sporulation as environmental sodium chloride concentrations increase. In this project, we tested whether increased sodium chloride concentrations increase rates of *S. paradoxus* meiosis.

Contrary to our expectation, salt decreased *S. paradoxus* sporulation in a laboratory experiment. We cultured sixteen *S. paradoxus* strains, previously isolated from a forest in Germany, in growth medium with each of three salt concentrations: 0%, 3%, and 6%. We chose these salt concentrations because they affected a different *Saccharomyces* phenotype, sensitivity to killer toxins, in a different study (Llorente et al. 1997). We grew cells in standard growth media supplemented with sodium chloride (Table 2) for seven days. This long incubation allowed *S. paradoxus* cells to deplete carbon and nitrogen in the environment and begin to undergo meiosis. We scored cultures for sporulation frequency from photographs taken under a microscope. Sporulation decreased from a mean of 90% sporulated cells with no added salt to a mean of 67% with 6% salt (Z = -8.5, p < 10 ^-15^ , marginal R ^2^_GLMM_ = 0.46, Figure 1B ).

We hypothesize that interactions among salt stress, mitochondrial function, and meiosis might explain why meiosis decreased as salt concentration increased. Mitochondrial function is necessary for activating the IME1 transcription factor, which induces meiosis (Treinin and Simchen 1992, Zhao et al. 2018). Osmotic stress has a variety of potential impacts on mitochondrial function: it is associated with changes in mitochondrial gene expression and accumulation of reactive oxygen species in mitochondria (Di Noia et al. 2023, Pastor et al. 2009). Some mutations in *S. cerevisiae* cells also result in decreased respiration with salt stress (Guaragnella et al. 2021), suggesting that salt can directly inhibit respiration. An alternative explanation for our observations is that salt stress increased mitosis rates while meiosis rates stayed the same, decreasing the relative number of sporulated cells we saw. We consider this unlikely because salt stress is associated with the Hog1 pathway, which halts the mitotic cell cycle (de Nadal and Posas 2022). Other impacts of salt on cell function, such as increasing glycerol accumulation or disrupting DNA replication, are more likely to increase meiotic frequency relative to mitosis (Cruz-León et al. 2022, Patel and Miller 1972), so we do not consider these responsible for the pattern we observed.

Our results suggest that, while sexual life cycles may be favored with nutrient stress, they are not favored in all stressful environments. This diversity in responses to stress could be a result of molecular interactions inside the cell, for example, in the mitochondrion; it also might reflect the diversity of ecological impacts of different stressors for individual yeast cells. The identities and importances of stressors *S. paradoxus* encounters in natural environments could explain its population-wide infrequent rate of meiosis (Tsai et al. 2008).

## Methods

We grew sixteen wild *S. paradoxus* isolates (Table 1) in each of three salt concentrations: no salt (0% NaCl), low salt (3% NaCl), and high salt (6% NaCl) (Table 2). All isolates had been isolated in 2017 or 2018 from soil or leaf litter next to oak trees in Nehmtener Forst, a mixed temperate forest in Nehmten, Schleswig-Holstein, Germany (Boynton et al. 2021). We inoculated each isolate from a colony on a petri dish into a single replicate of 1 mL of each medium type using a sterile stick and included uninoculated controls of each medium type to control for culture contamination. We incubated cultures at 26°C with 180 rpm (revolutions per minute) shaking. After seven days of incubation, we mixed each culture, pipetted 5 µl onto a microscope slide, and took a photograph under a compound microscope. We scored sporulation frequencies by drawing a transect on each photograph and counting sporulated and nonsporulated cells ( Figure 1A ), up to 100 total cells, along each transect. Spores are observed as four haploid cells inside an ascus formed during sporulation; non-spores remain in their vegetative state as single or budding cells ( Figure 1A ). Sporulation proportion is equal to the number of sporulated cells divided by 100 (the total number of counted cells).

We modeled proportions of cells sporulated as a function of salt concentration using a generalized mixed-effects model, modeling variance in sporulation using the binomial family with a logit link function; we varied slopes and intercepts for each *S. paradoxus* isolate to account for the repeated-measures design of our experiment. Marginal R ^2^_GLMM_ ( *i.e.,* proportion of variance in sporulation explained by salt concentration) (Nakagawa et al. 2017) was calculated using the theoretical method of determining binomial observation errors. Statistical analyses were done using R version 4.5.2 (R Core Team, 2025) and the tidyverse, lme4, AICcmodavg, and MuMIn packages (Bartón 2025, Bates et al. 2015, Mazerolle 2023, Wickham et al., 2019).

## Reagents

Table 1: Fungal strains

**Table:** 

Species	Strain	Reference
*S. paradoxus*	5056	Boynton et al. 2021
*S. paradoxus*	5057	Boynton et al. 2021
*S. paradoxus*	5062	Boynton et al. 2021
*S. paradoxus*	5069	Boynton et al. 2021
*S. paradoxus*	5074	Boynton et al. 2021
*S. paradoxus*	5078	Boynton et al. 2021
*S. paradoxus*	5080	Boynton et al. 2021
*S. paradoxus*	5081	Boynton et al. 2021
*S. paradoxus*	5082	Boynton et al. 2021
*S. paradoxus*	5093	Boynton et al. 2021
*S. paradoxus*	5135	Boynton et al. 2021
*S. paradoxus*	5144	Boynton et al. 2021
*S. paradoxus*	5165	Boynton et al. 2021
*S. paradoxus*	5167	Boynton et al. 2021
*S. paradoxus*	5185	Boynton et al. 2021
*S. paradoxus*	5202	Boynton et al. 2021

Table 2: Growth media

**Table:** 

Medium	Composition
YPD 0% salt	10 g/L yeast extract, 20 g/L peptone, and 20 g/L dextrose
YPD 3% salt	10 g/L yeast extract, 20 g/L peptone, 20 g/L dextrose, and 30 g/L sodium chloride
YPD 6% salt	10 g/L yeast extract, 20 g/L peptone, 20 g/L dextrose, and 60 g/L sodium chloride

## Data Availability

Description: Sporulation data. Resource Type: Dataset. DOI: https://doi.org/10.22002/b0mff-2ex45
